# Dissemination of Registered COVID-19 Clinical Trials (DIRECCT): a cross-sectional study

**DOI:** 10.1186/s12916-023-03161-6

**Published:** 2023-11-29

**Authors:** Maia Salholz-Hillel, Molly Pugh-Jones, Nicole Hildebrand, Tjada A. Schult, Johannes Schwietering, Peter Grabitz, Benjamin Gregory Carlisle, Ben Goldacre, Daniel Strech, Nicholas J. DeVito

**Affiliations:** 1 QUEST Center for Responsible Research, Berlin Institute of Health (BIH), Charite Universitätsmedizin Berlin, Berlin, Germany; 2 Bennett Institute for Applied Data Science, Nuffield Department of Primary Care Health Sciences, University of Oxford, Oxford, UK

**Keywords:** Clinical trials transparency, COVID-19, Research integrity, Clinical trials, Results reporting

## Abstract

**Background:**

The results of clinical trials should be completely and rapidly reported during public health emergencies such as COVID-19. This study aimed to examine when, and where, the results of COVID-19 clinical trials were disseminated throughout the first 18 months of the pandemic.

**Methods:**

Clinical trials for COVID-19 treatment or prevention were identified from the WHO ICTRP database. All interventional trials with a registered completion date ≤ 30 June 2021 were included. Trial results, published as preprints, journal articles, or registry results, were located using automated and manual techniques across PubMed, Google Scholar, Google, EuropePMC, CORD-19, the Cochrane COVID-19 Study Register, and clinical trial registries. Our main analysis reports the rate of dissemination overall and per route, and the time from registered completion to results using Kaplan–Meier methods, with additional subgroup and sensitivity analyses reported.

**Results:**

Overall, 1643 trials with completion dates ranging from 46 to 561 days prior to the start of results searches were included. The cumulative probability of reporting was 12.5% at 3 months from completion, 21.6% at 6 months, and 32.8% at 12 months. Trial results were most commonly disseminated in journals (*n* = 278 trials, 69.2%); preprints were available for 194 trials (48.3%), 86 (44.3%) of which converted to a full journal article. Trials completed earlier in the pandemic were reported more rapidly than those later in the pandemic, and those involving ivermectin were more rapidly reported than other common interventions. Results were robust to various sensitivity analyses except when considering only trials in a “completed” status on the registry, which substantially increased reporting rates. Poor trial registry data on completion status and dates limits the precision of estimates.

**Conclusions:**

COVID-19 trials saw marginal increases in reporting rates compared to standard practice; most registered trials failed to meet even the 12-month non-pandemic standard. Preprints were common, complementing journal publication; however, registries were underutilized for rapid reporting. Maintaining registry data enables accurate representation of clinical research; failing to do so undermines these registries’ use for public accountability and analysis. Addressing rapid reporting and registry data quality must be emphasized at global, national, and institutional levels.

**Supplementary Information:**

The online version contains supplementary material available at 10.1186/s12916-023-03161-6.

## Background

Complete and timely reporting of trials allows for evidence to rapidly translate into clinical practice — this need is more acute during public health emergencies. The World Health Organization (WHO) recommends that dissemination of trial results in a global health emergency should be “greatly shortened” from the usual expectation of 12 months [1], although this has not occurred in past pandemics with many trials failing to meet even non-pandemic expectations [2]. Since 2020, the number of registered clinical trials addressing COVID-19 grew alongside the pandemic: the WHO International Clinical Trials Registry Platform (ICTRP) database went from 240 COVID-19 trial registrations in February 2020 to 20,076 on 5 May 2023 [3].

Clinical trial registration and results reporting are moral, ethical, pragmatic, and often legal requirements [4–7] and should ensure a public accounting of planned, ongoing, and completed clinical trials. Due to these various mandates, trial registries have become an essential piece of public health infrastructure and a valuable tool for research. They provide an avenue for transparency and accountability by making the planned methods, timeline, and outcomes of a trial public. Expectations are then set for when results should be available and what they should contain. Some registries also have the ability to directly host results. This robust system of registration and reporting compliments and informs dissemination in other fora like journals and preprints [8] and can aid in evidence synthesis [9], landscape assessments [10], and planning future research [11].

The primary objective of this study was to evaluate results reporting of COVID-19 trials completed in the first 18 months of the pandemic (i.e., January 2020 through June 2021). It leverages clinical trial registry data to examine a comprehensive population of COVID-19 research. This allows tracking of where and when the dissemination of clinical trials occurred during the pandemic. These results can help inform guidance on how research should be managed, reported, coordinated, and synthesized during future emergency situations. These findings extend on our previously published interim results on trials completed during the first 6 months of the pandemic, in which we found that 14% (41/285) of trials rapidly reported results and that preprints were most commonly used [12].

## Methods

The DIRECCT project is a meta cross-sectional study that examined the availability of results of trials completed through the first 18 months of the COVID-19 pandemic (i.e., January 2020–June 2021). Further details on the methods are available on the Open Science Framework in a preregistered protocol (https://doi.org/10.17605/OSF.IO/8FR9T) and an updated protocol inclusive of all amendments (https://doi.org/10.17605/OSF.IO/5F8J2). This study was reported according to the STROBE checklist for cross-sectional studies (Additional File 1) [13].

### Trial population

We used the WHO ICTRP list of registered COVID-19 trials [14], updated through 1 July 2021, as our primary data source. This was supplemented with additional data collected directly from clinical trial registries. This allowed access to information not present in the ICTRP dataset and better management of duplicate registrations. Cross-registrations (i.e., a trial with multiple registrations) were identified in registry data and results publications and collapsed into a single record prior to final analysis.

### Inclusion and exclusion criteria

We aimed to include all trials, of any design, examining an intervention for the treatment or prevention of COVID-19 infection and acute disease. The inclusion and exclusion criteria remained consistent with those in the interim report of the study [12], including the previously declared post hoc additions (Table 1). Existing determinations on exclusions from our preliminary analysis were carried forward into this final analysis.

**Table 1:** Inclusion and exclusion criteria

**Inclusion criteria:**
• Trial assessed an intervention for efficacy in the treatment or prevention of COVID-19 infection and subsequent acute disease.^a^
• Trial included a completion date, or primary completion date, on or before 30 June 2021
**Exclusion criteria:**
• Registration was found, at any time, to indicate that the trial was withdrawn before enrollment and therefore never occurred
• Registration prior to 1 January 2020
• Trial exclusively on symptomatic treatment of COVID-19 disease side-effects only (post hoc)
• Trial on rehabilitation after acute disease or treatment of post-COVID-19 disease (post hoc)

^a^During final data collection, two main clarifications were necessary for this criteria: First, we excluded trials with primary outcomes that examined only the safety of an intervention; Second, studies examining inflammatory biomarkers (e.g., D-Dimer, C-Reactive Protein) were counted as efficacy outcomes

Inclusion and exclusion criteria were applied first automatically using data extracted from the ICTRP and individual registries. This screened out observational studies, those in a “withdrawn” status, and those registered prior to 2020 or completed after the initiation of our searches. For all trials passing this automated step, inclusion and exclusion criteria were then manually assessed prior to manual searches for trial results within the Numbat Systematic Review Manager.

### Data extraction

For automated trial results searches, PubMed metadata was extracted for all records matching a PRESS Peer Reviewed search strategy for COVID clinical trials from the COVID-evidence project [15]. Trial IDs from the WHO ICTRP list of registered COVID-19 trials were text searched in the PubMed metadata as well as in the free-text of the CORD-19 database of open access coronavirus-related literature. Potential hits were presented to searchers and reviewed during the manual search process.

The interventions studied in included trials were extracted from trial registries. A “unique” study arm was recorded if it differed from other arms in either the intervention(s) used or the dosing, but not if it differed only in the population (e.g., age cohorts) receiving the same intervention. Interventions under study were separated from controls and standards of care given in all arms. Interventions were then manually reviewed and normalized, grouped, and deduplicated, regardless of dose, to arrive at the unique interventions used in each trial.

### Search strategy

Following manual inclusion screening, trials progressed to manual searches. First, the trial’s registrations and any automatically identified potential publications from PubMed or CORD-19 were screened for any published results. Keyword searches were then conducted in the Cochrane COVID-19 study register, PubMed, Europe PMC, Google Scholar, and Google. Search terms covered the trial IDs, interventions, investigators, sponsor, and any other relevant or distinct keywords at the discretion of the searcher.

We had planned to separately examine reporting for the first 12 and 18 months of the pandemic. However, due to the large volume of COVID-19 trials, both analyses were collapsed into a final 18-month analysis. Manual searches took place between March 2021 and January 2022; each trial was searched at least once after 15 August 2021 to ensure a minimum of at least 6 weeks had passed from any given trial’s registered completion date. Additional validations and checks were made to the dataset during cleaning, preparation, and analysis through January 2023.

### Data validation

As the number of COVID-19 trials was much larger than our initial expectations, searches in duplicate for all trials were not possible with available resources. In light of this, a number of strategies were adopted to minimize bias and extraction errors. All trials completed through 31 December 2020 (i.e., the first 12 months) were searched independently, in duplicate, with at least one search occurring after the final results cut-off date of 15 August 2021. For the remaining trials, any issues experienced by extractors during solo-extraction were flagged and triggered an independent second search by another team member. All trials manually screened for inclusion and searched in duplicate were reconciled by consensus between extractors, and any remaining issues were referred to the study leadership team (MSH, NJD) for final adjudication. Notable edge cases in our population are detailed in Additional File 2: Appendix A. Following the completion of data extraction, preprint-journal article matches were validated using the Bio/MedRxiv API [16] when possible, and the leadership team manually reviewed all remaining combinations to ensure correct matching and categorization.

### Outcomes

#### Outcome definitions

Searchers recorded any trial results disseminated as a journal publication, preprint publication, or stand-alone results on a registry. In addition, journal and preprint publications were assessed as being “interim” or “full.” Full trial results were those that contained the complete follow-up for at least one primary outcome. Publications were matched to registrations, and to each other (i.e., preprints to publications) by comparing titles, interventions, investigators, and basic design characteristics with ambiguities referred for adjudication. Registry results had to be hosted on the registry, rather than simply link to an external results document or paper, and meet the minimum ICTRP standard for summary trial results to be counted (i.e., contain baseline characteristics, participant flow, adverse events, and outcome measures) [17].

#### Trial results reporting

Per protocol, our primary analysis is based on registry data as it stood when collected in early July 2021. When a trial was cross-registered on multiple databases, we took key information, like completion dates, from the registry with the most recent update, or from the EU Clinical Trial Register (EUCTR) which only includes completion dates after a trial completes. We report summary statistics on trial results availability across any route, and for each individual route. Additionally, we generated cumulative incidence curves using Kaplan–Meier methods with unreported trials censored on 15 August 2021. For the time to preprint publication model, the cumulative incidence curve was fit using the Aalen-Johansen method with journal publication as a competing risk and ties broken by nominal offsets [18]. For time to registry results, we limited our population to trials with a registration on ClinicalTrials.gov, the EUCTR, or the ISRCTN, as these registries have the most mature processes for hosting results meeting our definition. Trials with results published prior to the registered completion date were considered reported at time zero. We additionally investigated time from preprint to journal publication using Kaplan–Meier methods.

#### Subgroup analyses

Time-to-report comparisons, using cumulative incidence curves, were also generated for various sub-populations. These additional analyses were modified from, or added post hoc to, the original protocol. First, we examined how trial reporting timelines changed over time. Trials were stratified into three 6-month periods covering the first 18 months of the pandemic (i.e., January 2020–June 2021) based on when they completed. These 6-month periods align with the original prespecified analysis plan. Next, cumulative incidence curves for studies containing each of the five most common interventions were fit. Lastly, we restricted our sample to those meeting certain design characteristics and enrollment standards, as a proxy for those most likely to influence clinical practice. We defined these as late-phase (i.e., ≥ Phase 2), randomized trials enrolling at least 100 participants. Trial characteristics were extracted from the ICTRP dataset using previously validated automated methods [19].

#### Sensitivity analyses

Four sensitivity analyses, all post hoc, were conducted in order to check whether reporting rates were sensitive to changes in methods. Each sensitivity analysis was applied independently, not cumulatively. First, only full study completion dates were used, rather than primary completion dates when available. Next, the final population was restricted to only those that had both reached their registered completion date and updated their trial registration to a “completed” status, indicating proactive acknowledgement the trial took place and completed. We then expanded our definition of “first results” to include interim results that did not include complete follow-up of a primary outcome. Lastly, we re-extracted data from all registries in April 2022 and applied these updated completion and trial status data retrospectively to our sample for re-analysis. For each sensitivity analysis, we examined the raw reporting rates and cumulative incidence curves, and compared them to our primary findings.

### Software, data, and code

Data analysis was conducted in R V.4.3.0 (R Foundation for Statistical Computing, Vienna, Austria) and Python V.3.8.1 (Python Software Foundation, Wilmington, Delaware USA). Manual data extraction and reconciliation was conducted in Numbat Systematic Review Manager [20]. Code and data are available on Github [21, 22] and Zenodo [23].

## Results

**Fig. 1:**
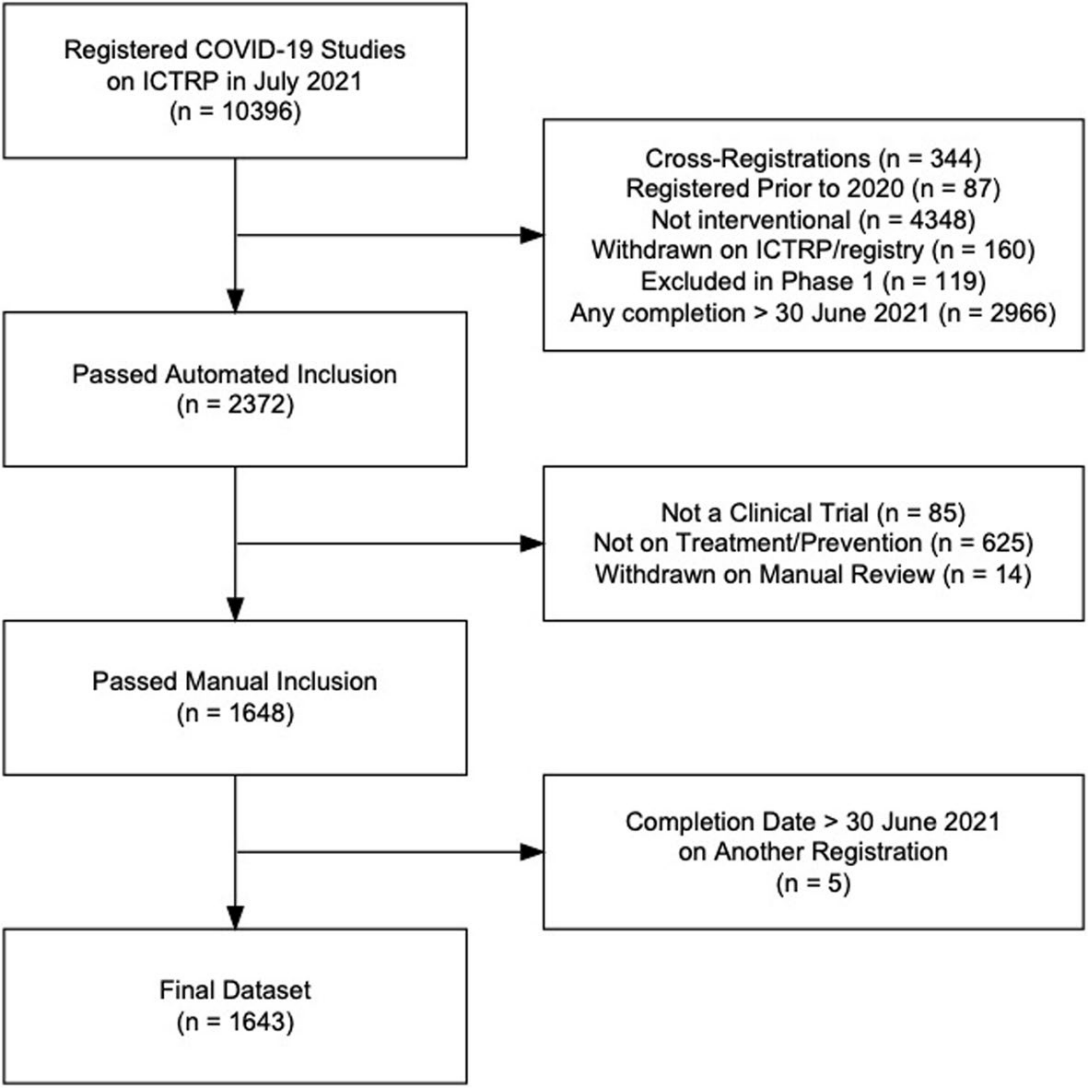
Flow-chart for trial inclusion. ICTRP, International Clinical Trials Registry Platform. Cross-registrations include those identified in automated and manual screening of registries and publications

As of 30 June 2021, the ICTRP COVID-19 database contained 10,396 interventional and observational clinical study registrations. After automated screening, and accounting for cross-registrations, 2372 completed interventional trials remained. After manual screening, 1643 completed interventional trials meeting our inclusion criteria were manually searched for our final analysis; 68% (*n* = 1,100) were searched by at least two investigators. A flow-chart detailing all exclusions is available in Fig. 1. Characteristics of the 1643 trials are included in Table 2.

**Table 2:** Characteristics of included trials, overall and subsetted by trials with and without results reported by the start of result searches 15 August 2021. Cross-registrations refer to registrations in 2 or more registries; multiple registrations of the same trial in the same registry are not counted as cross-registrations. Top interventions refer to most common individual interventions. The “Other” trial status includes trials in “Ongoing” and any other non-completed statuses

**Characteristic**	**Overall**^**a**^ *N* = 1643	**Trials with results**^**a**^ *N* = 402	**Trials without results**^**a**^ *N* = 1241
**Target enrollment**	100 (48, 253)	106 (43, 291)	100 (50, 250)
**Cross-registered**	205 (12%)	53 (13%)	152 (12%)
**Multinational**	102 (6.2%)	32 (8.0%)	70 (5.6%)
**Randomized**	1313 (80%)	311 (77%)	1002 (81%)
**Trial phase**			
Not applicable^b^	461 (28%)	122 (30%)	339 (27%)
Phase 1	78 (4.7%)	15 (3.7%)	63 (5.1%)
Phase 1/Phase 2	77 (4.7%)	30 (7.5%)	47 (3.8%)
Phase 2	429 (26%)	89 (22%)	340 (27%)
Phase 2/Phase 3	129 (7.9%)	29 (7.2%)	100 (8.1%)
Phase 3	306 (19%)	82 (20%)	224 (18%)
Phase 3/Phase 4	7 (0.4%)	1 (0.2%)	6 (0.5%)
Phase 4	156 (9.5%)	34 (8.5%)	122 (9.8%)
**Trial status**			
Completed	472 (29%)	206 (51%)	266 (21%)
Terminated	81 (4.9%)	24 (6.0%)	57 (4.6%)
Other	1090 (66%)	172 (43%)	918 (74%)
**Pandemic phase**			
Jan 2020–Jun 2020	282 (17%)	102 (25%)	180 (15%)
July 2020–Dec 2020	672 (41%)	197 (49%)	475 (38%)
Jan 2021–Jun 2021	689 (42%)	103 (26%)	586 (47%)
**Top 5 interventions**			
Hydroxychloroquine	138 (35%)	41 (33%)	97 (36%)
Convalescent plasma	118 (30%)	38 (30%)	80 (29%)
Stem cells (mesenchymal)	50 (13%)	11 (8.8%)	39 (14%)
Azithromycin	46 (12%)	13 (10%)	33 (12%)
Ivermectin	46 (12%)	22 (18%)	24 (8.8%)

^a^Target enrollment: median (IQR). All other characteristics: *n* (%)

^b^ “Not applicable” phases generally, though not exclusively, refer to non-drug trials on registries like ClinicalTrials.gov

### Trial results reporting

#### Registration of COVID-19 clinical trials

Trials were most commonly registered in ClinicalTrials.gov, followed by the Chinese Clinical Trial Registry. Overall, 210 trials (13%) had two or more registry entries that required deduplication. Reporting rates across registries were comparable, with trials on the ISRCTN most likely to have results across all dissemination routes (62%). Additional File 2: Figure S1 shows an upset plot of cross-registrations, and Additional File 2: Table S1 shows the reporting rate per registry without deduplication of records.

#### Dissemination of COVID-19 clinical trials

**Fig. 2:**
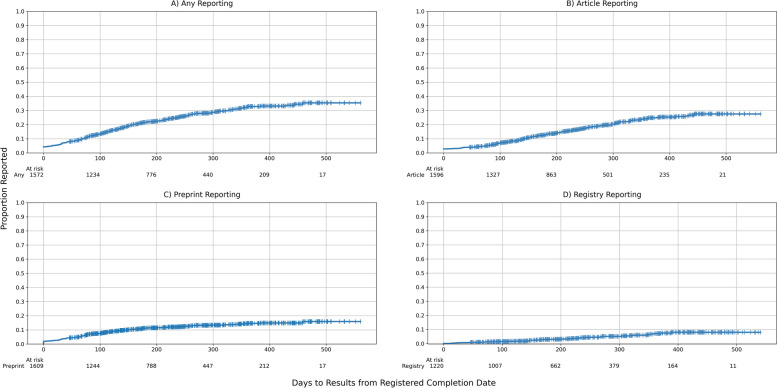
**A**–**D** Time to trial results reporting across dissemination routes. Summary results in the registry limited to the three registries with structured summary results reporting formats (EU Clinical Trial Register, ClinicalTrials.gov, ISRCTN). The Aalen-Johanssen plot of time to preprint publication (**C**) used nominal offsets to break ties at 0. Trials with a publication date prior to the available completion date (across all results, *n* = 71 of 402 trials, 18%) were considered reported at time 0

The cumulative probability of reporting under pandemic conditions was 12.5% at 3 months since completion, 21.6% at 6 months, and 32.8% at 12 months — in other words, just under a third of trials met the WHO’s normal standard for first dissemination of results, ideally on a trial registry, in non-emergency situations. The minimum time from completion was 46 days (i.e., six full weeks through 15 August 2021) with a maximum time of 561 days; median time from completion to searches was 250 days (IQR 138–369) and the median time to reporting was undefined for all models. Figure 2A–D shows cumulative incidence plots for time-to-publication for (a) first publication across any dissemination route, (b) earliest journal publication, (c) earliest preprint publication, and (d) summary results limited to the three registries with mature structured summary results reporting formats (i.e., EUCTR, ClinicalTrials.gov, ISRCTN; see Additional File 2: Fig. 2 for data across all registries). Overall, 402 trials (24%) had a result available prior to the start of searches spread across 545 individual results publications.

#### Dissemination routes for COVID-19 results

**Fig. 3:**
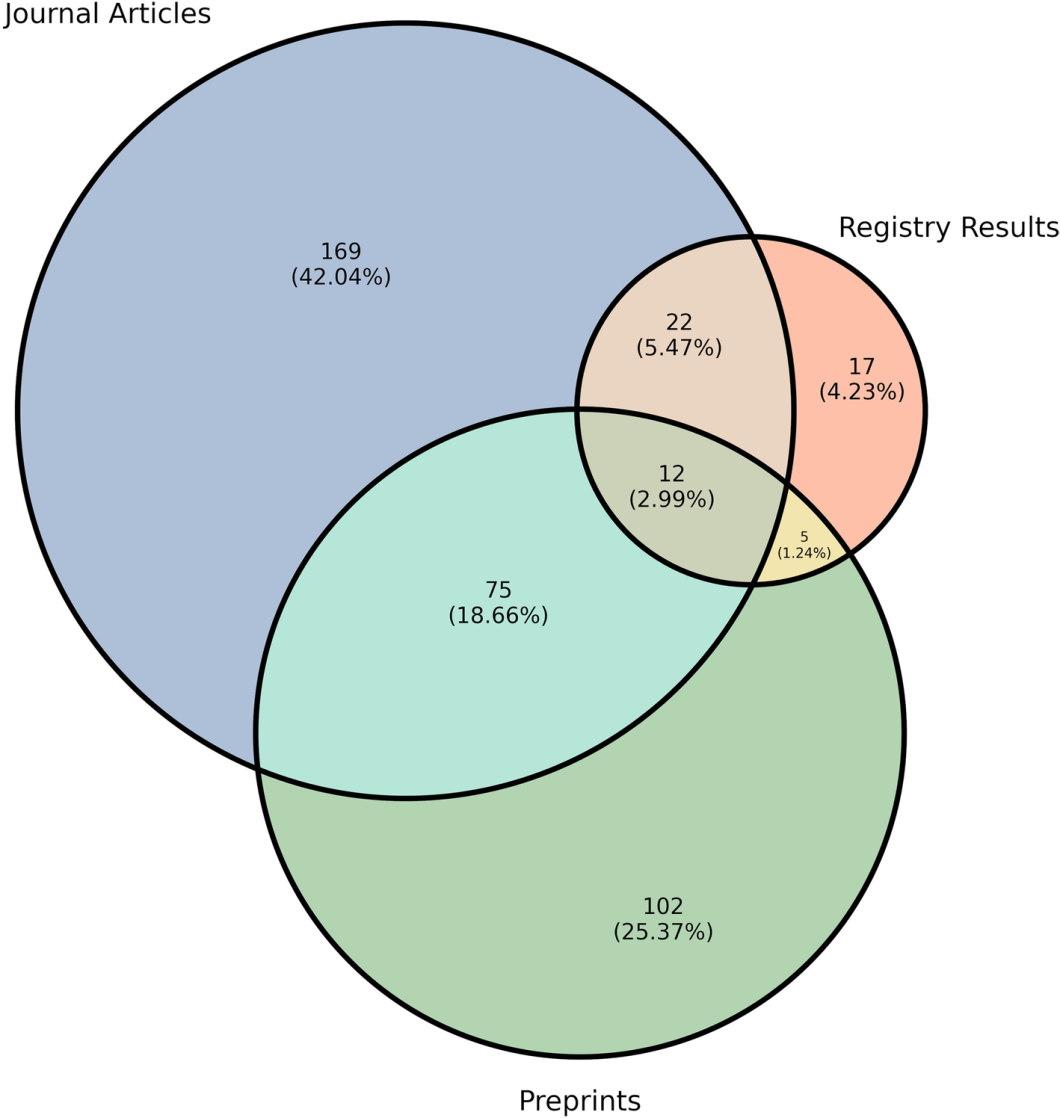
COVID-19 clinical trials with results by dissemination route. Trials reporting both a preprint and a full article include some non-matches, i.e., a preprint reporting full results of one primary outcome measure and an article reporting full results of a different primary outcome measure, which are both counted as full results

**Fig. 4:**
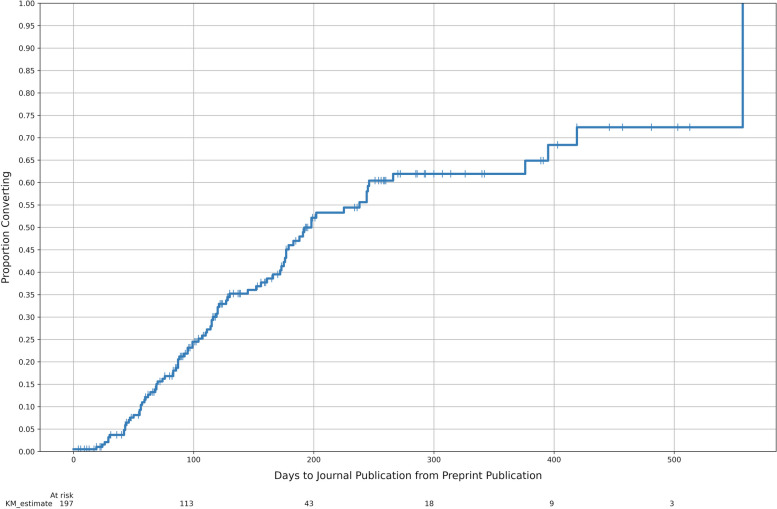
Time from preprint to journal publication

Of the 402 trials with results, journal articles were the most common dissemination route with 278 trials (69.2%) having a peer-reviewed publication reporting a primary endpoint. Reporting across all dissemination routes is detailed in Fig. 3. A primary result was available in a preprint for 194 trials (48.3%); matching preprint-journal pairs were located for 86 trials (21.4%), with one trial excluded from this count, as it had a preprint and journal article that were not matched as they reported different results from the same trial. When multiple preprint-article pairs (*n* = 1) or multiple preprints for a single article (*n* = 4) were located, we used the earliest preprint date in all analyses. Figure 4 shows the delay from preprint to full journal publication for all preprints; journal articles published prior to preprints were set to time 0, and preprints without a journal publication were censored at the start of result searches. The median time from preprint publication to journal publication was 198 days.

### Subgroup analyses

**Fig. 5:**
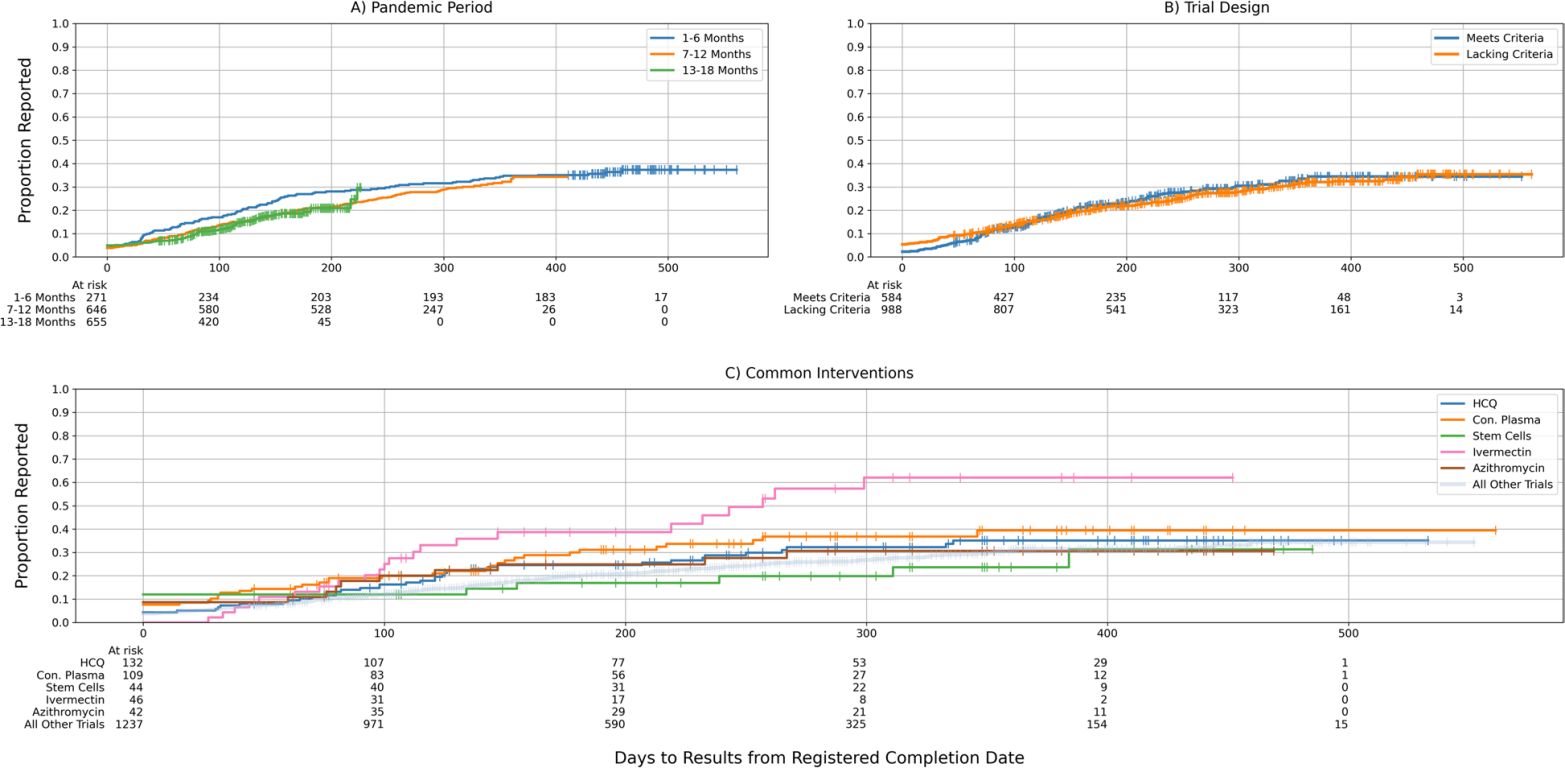
**A–C** Time to dissemination by subgroups. **A** 6-month phase of the pandemic. **B** Top 5 common interventions. **C** Based on trial design standards. HCQ: Hydroxychloroquine; Con. Plasma: Convalescent Plasma

Figure 5a shows time to reporting stratified by whether trials completed in the early, middle, or late periods of the first 18 months of the COVID-19 pandemic. Trials completed in the first 6 months of the pandemic were consistently reported sooner than those in the later parts of the pandemic at both 100 (Early: 17.0%; Mid: 13.6%; Late: 11.9%) and 200 days (Early: 28.0%; Mid: 21.4%; Late: 20.8%) from trial completion.

To investigate whether registered trial design characteristics influenced reporting, we separated all trials that were Phase 2 or higher, randomized, and enrolled at least 100 participants from those that did not have these characteristics. Trials were included if any registration indicated these characteristics were met. Of the 598 trials with these characteristics, 138 (23%) reported. Figure 5b shows how this compares to trials without these characteristics.

### Sensitivity analyses

First, limiting our sample to trials that had reached full completion by 30 June 2021 showed 374 of 1420 (26%) trials reported by the start of searches. Next, restricting our sample to trials with a “Completed” status (e.g., Completed, Terminated) reduced our sample to 553 trials, of which 230 (42%) reported by the start of searches. Then, expanding our results to interim findings added 68 results publications for a reporting percentage of 27% (*n* = 439 trials). Finally, using the completion dates available on the registries in April 2022 changed the completion date for 272 trials of the 1648 (17%) trials that passed manual screening. Of these, 215 trials’ completion dates were made later whereas 57 trials’ completion dates were moved earlier. An additional 7 trials had a withdrawn status on a registry in April 2022 and were excluded. The final dataset using completion dates and trial status available in April 2022 comprised 1486 trials of which 392 (26%) trials reported by the start of our searches. Additional File 2: Figure S3 shows cumulative incidence curves for each sensitivity analysis compared to the primary analysis.

## Discussion

### Summary of results

Examining all trials registered as completed during the first 18 months of the COVID-19 pandemic yielded a 32.8% cumulative probability of reporting at 12 months, with just over two-thirds of trials failing to meet the WHO’s non-pandemic standard for first dissemination of trial results. The median time from trial completion to results searches was 250 days (range 46–561 days). Despite a rise in the use and popularity of preprints, especially early in the pandemic, the most common dissemination route was to publish only in a journal article. Clinical trial registries were, comparatively, rarely used for rapid dissemination. The overall reporting rate was robust to a number of sensitivity analyses; however, trials with a completed status on a registry, in addition to having passed their listed completion date, had a notably higher reporting rate. Reporting was most rapid during the first 6 months of the pandemic compared to the subsequent two 6-month periods. Ivermectin showed notably different reporting patterns compared to other top interventions (i.e., hydroxychloroquine, convalescent plasma, azithromycin, and stem cells).

### Findings in context

This study builds on our interim findings, and studies tracking COVID-19 trials from other groups [12, 24–26]. Accelerated trial result reporting was consistent in this expanded population, as 13.5% of studies continued to report within 100 days of completion. As would be expected, more time to report led to an increase in overall trial results availability from 14 to 24%. While our preliminary findings showed a slight preference for preprints at the start of the pandemic, by the start of our searches on 15 August 2021, journal publications were the most common dissemination route. Still, the rise in the use of preprints remains substantial and notable, with 57% of reported trials in our population having a preprint available. However, the majority of preprints in our cohort remained unconverted into journal articles (55%, 111/202). Other research has shown concordance in reporting characteristics among COVID preprints that do convert to journal articles [27–29].

While the raw reporting rate of 24% is low, it does appear that results dissemination of completed trials was accelerated during the COVID-19 pandemic, both compared to prior pandemics and standard practice. Jones and colleagues examined reporting of trials for Ebola, H1N1, and Zika Virus, with time from completion ranging from ~ 18 months to ~ 72 months [2]. Only Ebola saw a journal publication rate exceeding 20% within a year from completion; the journal reporting rate for COVID-19 exceeded 20% within 300 days, and for any dissemination route in under 200 days. The delayed reporting found by Jones and colleagues is consistent with other findings from the H1N1 pandemic [30, 31]. Similar to our COVID-19 analysis, there was substantially lower dissemination on registries throughout these pandemics. Only five of 333 (1.5%) trials met the non-emergency WHO standard of having results on a registry within 12 months, and in a journal within 24 months; while 32.8% of COVID-19 trials had disseminated results within 12 months, only 7.2% reported on the registry, even when restricting the population to only the registries most likely to contain results. Based on the low usage of registries for rapid dissemination during the COVID-19 pandemic to date, compliance with this standard has not improved. In contrast with our findings, Jones and colleagues’ analysis did not find a noticeable change in overall reporting for trials in a completed status.

As in our interim findings, reporting of COVID-19 clinical trials appeared accelerated compared to standard practice. Other large studies examining the time to dissemination for clinical trials in non-pandemic situations show rates of dissemination within the first year far below the 32.8% seen in our findings [32, 33]. Even legally mandated reporting to ClinicalTrials.gov under US law leads to just 41% of trials reported within a year of primary completion [34]. These non-pandemic analyses, however, typically only cover journal articles and registry results; the rise of preprints may impact future analyses of time-to-publication should they continue to be used in non-COVID-19 contexts. However, even having one quarter of trials published in journals at 1 year would represent an improvement compared with recently documented practice [35–37].

In our assessment of common interventions, trials containing arms assessing convalescent plasma, hydroxychloroquine, and azithromycin showed reporting patterns similar to trials examining all other interventions outside of the top five most common. Stem cells also followed the same general trend though with slightly slower reporting. However, trials with an ivermectin treatment arm showed persistently more rapid reporting. This is notable given the serious concerns raised around both fraud and overall trial quality within ivermectin COVID-19 research [38, 39]. Also notable is the relatively low reporting rate of stem cell trials. Ivermectin and hydroxychloroquine, including its usage in combination with azithromycin, were the focus of intense attention, debate, and controversy during the pandemic [40–43]. While receiving less attention, convalescent plasma also garnered serious consideration as a potential treatment, including an emergency approval from the US Food and Drug Administration, before it was shown to be largely ineffective [44, 45]. However, stem cells were never elevated to similar levels of public, political, and media attention despite high apparent interest from the research community. This mismatch translating to the lowest, and slowest, reporting trends is a notable finding worthy of additional investigation.

### Strengths and limitations

This analysis presents a thorough overview of dissemination of clinical trial results during the COVID-19 pandemic. We are not aware of any other analysis that comprehensively examines the link between registration and publication of COVID-19 clinical trials across all ICTRP primary and data provider registries. We made efforts to limit duplication in our dataset through extensive checks for cross-registrations. Given that 13% of the trials in our final sample had multiple registry entries, failure to take this step could have likely impacted our conclusions. Our detailed documentation of these links between registrations and results across multiple dissemination routes could be a boon to future research examining COVID-19 clinical trials. As this is, to our knowledge, the largest comprehensive assessment of the reporting of COVID-19 clinical trials to date, our curated, open dataset can aid in making future metaresearch on the pandemic more efficient and complete.

We included all registered trials, not only randomized controlled trials, in this analysis as a reflection of the full scope of the COVID-19 research landscape. Other major COVID-landscape projects tended to focus on randomized trials, as they aimed to support evidence synthesis efforts [25, 46, 47]. Non-randomized studies, such as early research on hydroxychloroquine [48], were influential to the course of the pandemic despite their design limitations. A sensitivity analysis examining only late-phase, large, randomized studies was nearly identical to the overall reporting rate (23% vs. 24%).These samller, early-phase and non-randomized trials, though perhaps less influential for evidence synthesis and medical guidelines, represent the majority of our sample (64%, 1045/1643), and collectively enrolled thousands of participants at substantial overall cost, and thus have the same moral imperative to share timely results and avoid research waste.

While we could only search roughly two-thirds of our sample in duplicate and could not conduct outreach to investigators, due to resource constraints, our comprehensive search strategy ensured all trials underwent a thorough process for results discovery. Registries, COVID-19-specific study databases, and numerous bibliographic databases were searched using both automated and manual methods. In our efforts to be as inclusive as possible, we included non-English-language results, if we could reasonably translate or otherwise validate the connection to a given registration, though we recognize that the study team was not necessarily well positioned to locate results outside of their native languages and may have missed some results due to this limitation. Publications in non-English languages should still ideally include reference to the trial registration ID in the abstract and full-text which can help mitigate these discovery issue. Searchers were also encouraged to flag trials for adjudication and duplicate coding when faced with any doubts or questions.

This study aimed to examine the rapid dissemination of trial results within pandemic conditions leading to shorter time from completion to results searches than is typical for similar studies of trial non-publication. This approach allowed for feedback on pandemic trial reporting trends faster than typical retrospective analyses which usually occur years later. However, some studies crucial to the pandemic response, but with very long follow-up time, such as adaptive trials and vaccine trials, were not included in our population, as they remain ongoing with only interim results potentially available. We hope future research will build on our dataset of COVID-19 registration and publication through expanded and updated searches, to further understand how dissemination practices may have influenced clinical decision-making during the entirety of the COVID-19 pandemic.

The main limitation of this work was that poor data quality on clinical trial registries may have influenced our findings. Given existing concerns about the reliability of trial information across multiple registries [49], we took efforts to ensure we used more recent and complete data from across multiple registries when possible. We also attempted to examine the impact of data quality and found that using more recent data did not improve reporting statistics. However, the registry entries with more accurate upkeep, in the form of proactive updates to the trial status, did show markedly increased dissemination: the overall reporting rate nearly doubled (24% vs. 42%) when trials were limited to those that had proactively updated their trial to a “completed” status, in addition to having met their completion date.

Poor registry data could impact this analysis in a number of ways. First, the status of trials may be incorrect, resulting in the misclassification of ongoing, completed, terminated, and withdrawn trials. Trials that terminate early with partial enrollment are still expected to update their registrations and indicate whether they were (1) withdrawn prior to enrolling participants, and therefore no results could exist, or (2) terminated early after enrolling some participants. Terminated trials are still expected to report in some form, though reporting rates of these trials are known to be low [49–52]. Next, completion dates could be incorrect, leading to imprecision in reporting timelines and the potential for misclassification of “ongoing” studies. Our study showed such misspecification of completion dates on the registry: 71 trials, 18% of all results located, had results published on the same day or prior to the registered completion date. Refreshing our completion date data 10 months later did not make an appreciable difference to the overall reporting rate or trends, suggesting that increased time from trial completion does not see improved registry data quality. Lastly, proper maintenance of registry records is likely a positive predictor of trial reporting, which could be investigated in future research. While each of these mechanisms may play a role, better data on which trials actually occurred and when they completed would lead to more precise estimates of publication bias. We hope our open data can provide a starting point to further examine the impact of registry data quality on the validity of analyses of publication bias.

### Implications for policy and practice

Despite recommendations for accelerated reporting during public health emergencies, overall reporting remained low with most trials failing to meet even the non-pandemic 12-month standard for results dissemination on a clinical trial registry. The slight increase in reporting compared to standard practice, especially early in the pandemic, should not obscure the fact that more than two-thirds of all pandemic-relevant trials did not publish results within 12 months of the study end date on the registry. This is despite the rise in preprints to aid faster dissemination [50], the availability of registries to rapidly host results [51], and efforts by many journals and publishers to fast-track review of COVID-19 research [52]. Whether this lack of reporting is due to publication bias, a high number of aborted studies, or poor registration data, it underscores cause for concern.

Clinical trial registries cease to represent the current clinical trial landscape when they fail to present timely, accurate, and complete data. Evidence synthesis and research planning [9, 11] rely on registries to provide information on planned, ongoing, and completed trials. Neglecting registry data reduces the accuracy and efficiency of this work and threatens the quality of the resulting clinical guidelines and medical decision-making. COVID-19 was a unique global phenomenon and dominated the focus of new research. Unfortunately, as a result, it appears that in the rush to initiate new studies, many failed to start, ended early, or had difficulty with enrollment and simply abandoned their trials and registry entries [25]. As the high proportion of results discordant with registered completion dates show, even when studies unambiguously did occur, registries could not necessarily be counted on as accurate reflections of reality.

Similarly disappointing is that registries remain substantially underutilized as a rapid dissemination platform. Registries like ClinicalTrials.gov and the EUCTR have standard reporting formats that allow for the publication of results in parallel to preprint and journal publication. While the results have to meet some quality standards, there is no peer review, and no lead time for writing and formatting manuscripts, which should allow for more rapid dissemination. With new minimum standards for registry-hosted results under consultation at the ICTRP [53], registries will need to invest in encouraging and facilitating reporting, while researchers and their institutions should consider reporting to registries a routine aspect of results dissemination, especially during public health emergencies. Journal editors could also make registry maintenance and the posting of summary results a condition of publication, in much the same way they require prospective registration [54] and be more explicit that the publication of summary results on a registry does not count as prior publication, so as to encourage the use of registries as a complementary dissemination route.

While faster dissemination, via preprints or registries, does draw concerns around unvetted or low-quality results entering the public domain, it also allows high-impact results to be adopted into care more quickly [55]. Evidence has shown that COVID-19 preprints that convert to publications are typically concordant in their main characteristics [27, 28, 56] while those that remain unpublished tend to have more issues [29]. “Hot” topics like COVID-19 also likely draw more intense scrutiny during the pre-publication review process that will lead to public discussion around controversial or low-quality preprinted results [57]. The quality of results posted to ClinicalTrials.gov has consistently shown to be high when compared to journal publications for the same study [58–61].

Our results show that the many COVID-19 studies remain unpublished and have unclear registry data that hides their true status: stakeholders involved in clinical trials, including researchers, funders, registries, research institutions, ethics committees, and regulators, need to work together to facilitate timely publication and to ensure that registered data reflects a trial’s true status. Better coordination of emergency research among stakeholders can help to reduce the number of trials that terminated early due to false starts or failure to recruit [62, 63]. However, given low reporting rates and high uncertainty about the status of unreported trials, evidence synthesis efforts around COVID-19 treatments should routinely check for publication bias and make additional efforts to confirm the status of registered trials with investigators.

Governments and international bodies like the WHO should refine their guidance and laws around when and where results should be published, especially in public health emergencies. This will provide clear criteria that stakeholders should aim to achieve and that can be tracked and audited. Individual registries and coordinating bodies like the ICTRP should improve standards and processes for routine follow-up with trial sponsors to ensure data is updated and results clearly posted on, or clearly linked to, the registry. These efforts will reduce confusion and burden for future research planning, evidence synthesis, and metaresearch efforts. Aiming to improve these standards now will aid in ensuring that the knowledge infrastructure around future public health emergencies is better managed.

## Conclusion

Expectations to more rapidly report trial results during the COVID-19 public health emergency have not consistently led to more rapid reporting, as the vast majority of registered clinical trials still failed to meet this standard. Preprints were common during the pandemic, complementing journal publication as a method for dissemination; however, registries were not routinely used for rapid reporting. The importance of maintaining registry data, in order to provide an accurate representation of the research landscape, is a key issue that must be emphasized at the global, national, and institutional levels. Poor data quality also undermines the public purpose of clinical trial registration for public audit and analysis.

### Supplementary Information

**Additional file 1. **STROBE Statement—Checklist of items that should be included in reports of *cross-sectional studies*.

**Additional file 2. **Appendix A which details notable edge cases in our assessments for this project and Appendix B which contains supplementary figures and tables.

## Data Availability

The datasets generated and/or analyzed during the current study are available in the Zenodo repository (10.5281/zenodo.8181415) with code and additional data available via GitHub (https://github.com/maia-sh/direcct; https://github.com/ebmdatalab/direcct-phase2-python).

## Abbreviations

EUCTR: EU Clinical Trials Register
ICTRP: International Clinical Trials Registry Platform
WHO: World Health Organization
