# Supplementary material for: Dissemination of Registered COVID-19 Clinical Trials (DIRECCT): a cross-sectional study
Source: BMC Med. 2023 Nov 29;21:475. doi: 10.1186/s12916-023-03161-6 (PMC10687901; doi:10.1186/s12916-023-03161-6)
Supplement: Supplementary file 1 — Additional file 1. STROBE Statement—Checklist of items that should be included in reports of cross-sectional studies. [file 12916_2023_3161_MOESM1_ESM.docx]

STROBE Statement—Checklist of items that should be included in reports of ***cross-sectional studies***

|  | Item No | Recommendation |
| --- | --- | --- |
| **Title and abstract** | 1 | (*a*) Indicate the study’s design with a commonly used term in the title or the abstract  Cover Page |
|  |  | (*b*) Provide in the abstract an informative and balanced summary of what was done and what was found  Page 2 |
| Introduction | | |
| Background/rationale | 2 | Explain the scientific background and rationale for the investigation being reported  Page 6 |
| Objectives | 3 | State specific objectives, including any prespecified hypotheses  Pages 6-7 |
| Methods | | |
| Study design | 4 | Present key elements of study design early in the paper  Page 7 – Start of Methods Section |
| Setting | 5 | Describe the setting, locations, and relevant dates, including periods of recruitment, exposure, follow-up, and data collection  End of Page 9 |
| Participants | 6 | (*a*) Give the eligibility criteria, and the sources and methods of selection of participants  Page 8 |
| Variables | 7 | Clearly define all outcomes, exposures, predictors, potential confounders, and effect modifiers. Give diagnostic criteria, if applicable  Pages 11-13 |
| Data sources/ measurement | 8* | For each variable of interest, give sources of data and details of methods of assessment (measurement). Describe comparability of assessment methods if there is more than one group  Throughout Methods section |
| Bias | 9 | Describe any efforts to address potential sources of bias  Page 10 – Data Validation |
| Study size | 10 | Explain how the study size was arrived at  Study size is based on all trials meeting inclusion/exclusion criteria. Page 8. |
| Quantitative variables | 11 | Explain how quantitative variables were handled in the analyses. If applicable, describe which groupings were chosen and why  Page 11: Main analysis  Page 12: Sub-analyses and sensitivity analyses |
| Statistical methods | 12 | (*a*) Describe all statistical methods, including those used to control for confounding  Kaplan-Meir methods described on page 10. No other statistical comparisons made as descriptive |
|  |  | (*b*) Describe any methods used to examine subgroups and interactions  Page 12 |
|  |  | (*c*) Explain how missing data were addressed  Not Applicable – See Protocol |
|  |  | (*d*) If applicable, describe analytical methods taking account of sampling strategy  N/A |
|  |  | (*e*) Describe any sensitivity analyses  Pages 12-13 |
| Results | | |
| Participants | 13* | (a) Report numbers of individuals at each stage of study—eg numbers potentially eligible, examined for eligibility, confirmed eligible, included in the study, completing follow-up, and analysed  Page 13 and Figure 1 |
|  |  | (b) Give reasons for non-participation at each stage  Figure 1 |
|  |  | (c) Consider use of a flow diagram  Figure 1 |
| Descriptive data | 14* | (a) Give characteristics of study participants (eg demographic, clinical, social) and information on exposures and potential confounders  Table 1 |
|  |  | (b) Indicate number of participants with missing data for each variable of interest  N/A |
| Outcome data | 15* | Report numbers of outcome events or summary measures |
| Main results | 16 | (*a*) Give unadjusted estimates and, if applicable, confounder-adjusted estimates and their precision (eg, 95% confidence interval). Make clear which confounders were adjusted for and why they were included  Throughout Results. No models or statistical comparison so no need for confoundings. |
|  |  | (*b*) Report category boundaries when continuous variables were categorized  Table 1 |
|  |  | (*c*) If relevant, consider translating estimates of relative risk into absolute risk for a meaningful time period  Not applicable |
| Other analyses | 17 | Report other analyses done—eg analyses of subgroups and interactions, and sensitivity analyses  Pages 18-19 |
| Discussion | | |
| Key results | 18 | Summarise key results with reference to study objectives  Page 20 |
| Limitations | 19 | Discuss limitations of the study, taking into account sources of potential bias or imprecision. Discuss both direction and magnitude of any potential bias  Strengths and Limitations – Pages 23-25 |
| Interpretation | 20 | Give a cautious overall interpretation of results considering objectives, limitations, multiplicity of analyses, results from similar studies, and other relevant evidence  Throughout discussion starting on Page 20 |
| Generalisability | 21 | Discuss the generalisability (external validity) of the study results  Strengths and Limitations – Pages 23-25 |
| Other information | | |
| Funding | 22 | Give the source of funding and the role of the funders for the present study and, if applicable, for the original study on which the present article is based  Page 29 |

*Give information separately for exposed and unexposed groups.

**Note:** An Explanation and Elaboration article discusses each checklist item and gives methodological background and published examples of transparent reporting. The STROBE checklist is best used in conjunction with this article (freely available on the Web sites of PLoS Medicine at http://www.plosmedicine.org/, Annals of Internal Medicine at http://www.annals.org/, and Epidemiology at http://www.epidem.com/). Information on the STROBE Initiative is available at www.strobe-statement.org.
