# Supplementary material for: Dissemination of Registered COVID-19 Clinical Trials (DIRECCT): a cross-sectional study
Source: BMC Med. 2023 Nov 29;21:475. doi: 10.1186/s12916-023-03161-6 (PMC10687901; doi:10.1186/s12916-023-03161-6)
Supplement: Supplementary file 2 — Additional file 2. Appendix A which details notable edge cases in our assessments for this project and Appendix B which contains supplementary figures and tables. [file 12916_2023_3161_MOESM2_ESM.docx]

# Supplement

## Appendix A: Trial adjudication edge-cases

**ISRCTN44152751**: We excluded this trial based on a [tweet](https://web.archive.org/web/20200514164442/https://twitter.com/atlefretheim/status/1258725059071545346), found incidentally during Google results searches, by the trial lead which indicated that the trial was withdrawn prior to data collection. Per protocol, we generally considered information only from a registry or relevant publication to indicate that a trial did not meet our criteria (i.e., not a trial, completed after cut-off, etc.). We did not systematically search Twitter or secondary sources of data, such as reviews for trial information. However, we also aimed to exclude all trials found to have been “Withdrawn” meaning they never occurred. On balance, we determined that excluding a known withdrawn trial took priority in this instance, and we excluded the trial from our population. As of 5 June 2023, the trial registration had still not been updated to reflect the withdrawn status.

**EUCTR2020-001376-15/NCT04387409**: This trial has different completion dates within and across registrations: 1 May 2021 per ClinicalTrials.gov; 28 April 2021 per EUCTR summary results; 3 September 2020 per EUCTR registration (last checked: 5 June 2023). The summary results on EUCTR also indicate that the trial was prematurely terminated. While all dates are prior to our cutoff of 30 June 2021, the completion date impacts the time to publication. Per protocol, in our main analysis, we use the completion date in the EUCTR registration; in our sensitivity analysis preferring dates in results when available, we use the completion date in the EUCTR summary results, and thus exclude the trial from the population in that sensitivity analysis.

**EUCTR2020-001246-18/NCT04324047**: The CORIMUNO trial used a cohort multiple Randomized Controlled Trials (cmRCT) design. It comprised numerous “sub”-trial registrations all connected to an observational “supra”-trial. Each sub-trial included independent patient populations, so we considered each sub-trial as an individual trial (NCT04324073, NCT04345991, NCT04341584, NCT04343144, NCT04344756, NCT04344782, NCT04331808, NCT04346797, NCT04341870, NCT04476979). The supra-trial was also registered as EUCTR2020-001246-18 and NCT04324047.

**NCT04325906/NCT04347941/NCT04358939/NCT04395144/NCT04391140/NCT04477655**: Numerous prone positioning trials were combined and reported in a single pooled analysis, referred to as a “meta-trial”. This meant these trials were fully reported, but individually their data could not be ascertained from the main results publication. As such, we considered this meta-trial as, effectively, a large multi-centre trial and collapsed them to a single record in our dataset.

**ISRCTN15281137/NCT04324606**: The Oxford/AstraZeneca vaccine trial includes numerous trials which are reported in both stand-alone and pooled analyses. Upon close examination of the registrations and results publications, ISRCTN15281137 and NCT04324606 were found to refer to the same trial with the same participants and are thus counted as duplicate registrations. Other trials (ISRCTN89951424/NCT04400838/NCT04444674/NCT04516746/PACTR202005681895696) include different participants and are reported separately and thus are not collapsed into a single record.

**NCT04446429**: This key information within this trial registration, including the interventions, was changed numerous times. Numerous publications, describing different studies in different populations, were also linked to this registration. We used the information in the registration to match the publication of record in our dataset. Of note, this publication was subsequently [retracted](https://doi.org/10.3389/fmed.2022.964099); however we did not consider retraction in this study.

**ChiCTR2000030262**: The same registration was used as the registration of record for a pilot and full trial but did not differentiate between the two in the registration. We therefore considered the full trial for the full results. We considered 10.1016/j.eclinm.2020.100478 to be interim results, since it reports pilot and a primary outcome related to safety. We considered 10.1016/j.eclinm.2020.100547 to be full results, since reports results from main study and efficacy primary outcome.

**​​EUCTR2020-001113-21/ISRCTN50189673/NCT04381936**: The RECOVERY trial is an adaptive trial, meaning it is continuously opening and closing arms while enrolling new patients. The primary outcomes, however, are generally consistent across arms. Since we defined results to be interim until the publication of at least one primary outcome in *all* patients, all findings from the RECOVERY trial at the time of extraction results were coded as interim and excluded from our main analysis.

**EUCTR2020-000936-23/NCT04315948**: The DISCOVERY trial was designed in accord with, and provided data to, the multinational SOLIDARITY trial. DISCOVERY then went on to present its own independent analysis from just its study population. Since DISCOVERY independently reported results, and its other results could not be disaggregated from the results of SOLIDARITY, we counted DISCOVERY as its own trial with its own results.

## Appendix B: Supplemental Figures and Tables

###### **Supplemental Figure S1: Registration of COVID-19 Clinical Trials**


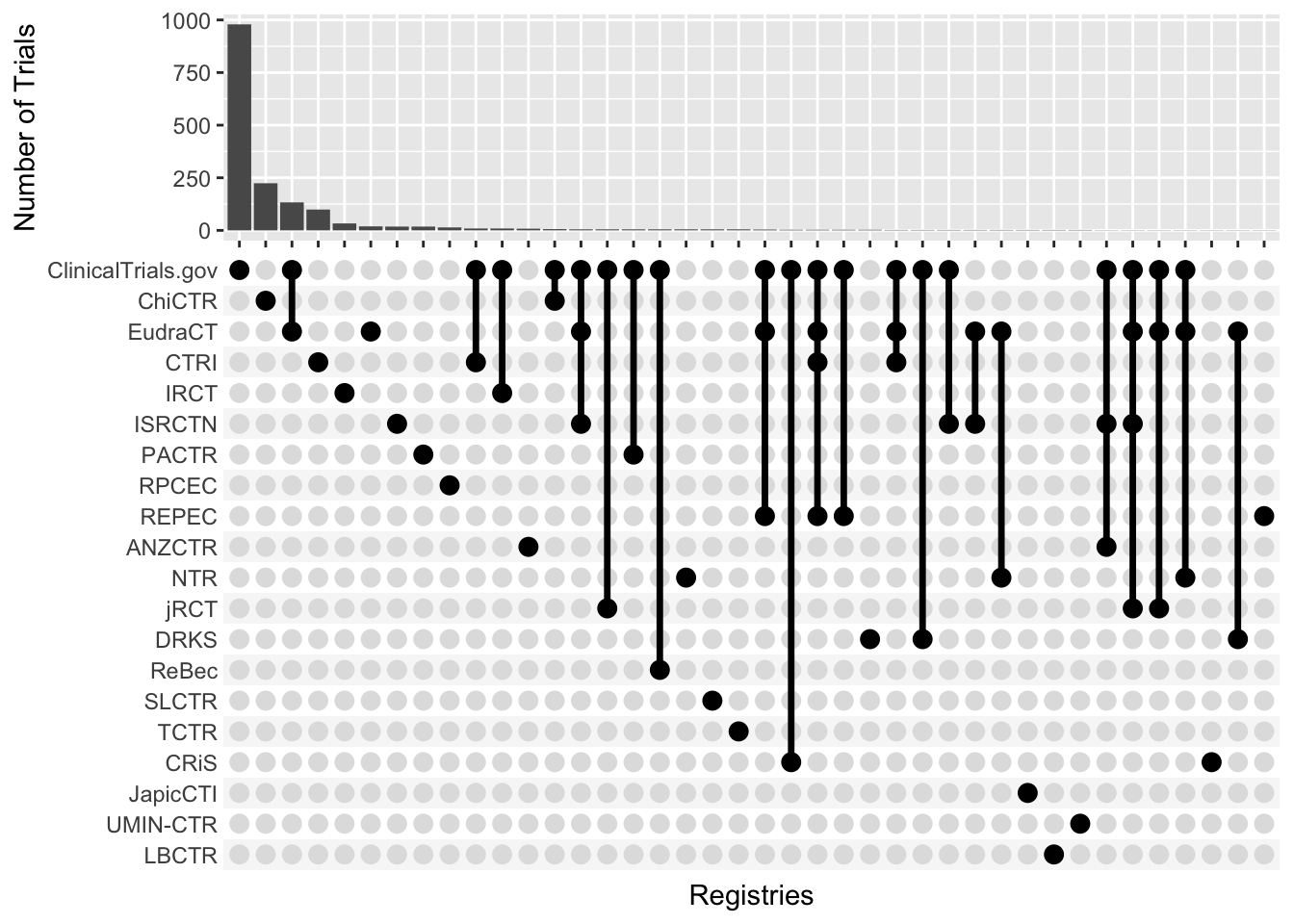


###### Supplemental Figure 1. Registrations of trials across registrations. Cross-registrations shown as intersections of registries.

######

###### **Supplemental Table S1: Reporting Rate by Registry**

| **Registry** | **Reporting Rate (%, n/N)** |
| --- | --- |
| ClinicalTrials.gov | 24.17% (285/1179) |
| ChiCTR | 23.04% (53/230) |
| EudraCT | 21.26% (37/174) |
| CTRI | 34.51% (39/113) |
| IRCT | 30.95% (13/42) |
| ISRCTN | 62.07% (18/29) |
| RPCEC | 35.71% (5/14) |
| REPEC | 18.18% (2/11) |
| ANZCTR | 11.11% (1/9) |
| NTR | 25.00% (2/8) |
| jRCT | 28.57% (2/7) |
| DRKS | 50.00% (3/6) |
| ReBec | 40.00% (2/5) |
| LBCTR | 50.00% (1/2) |
| UMIN-CTR | 50.00% (1/2) |

*Supplemental Table 1. Result reporting rate per clinical trial registry. Results include summary results on the registry, preprints, and journal articles. Records were not de-duplicated.*

###### **Supplemental Figure S2: Trial Reporting Across All Registries**

###### **
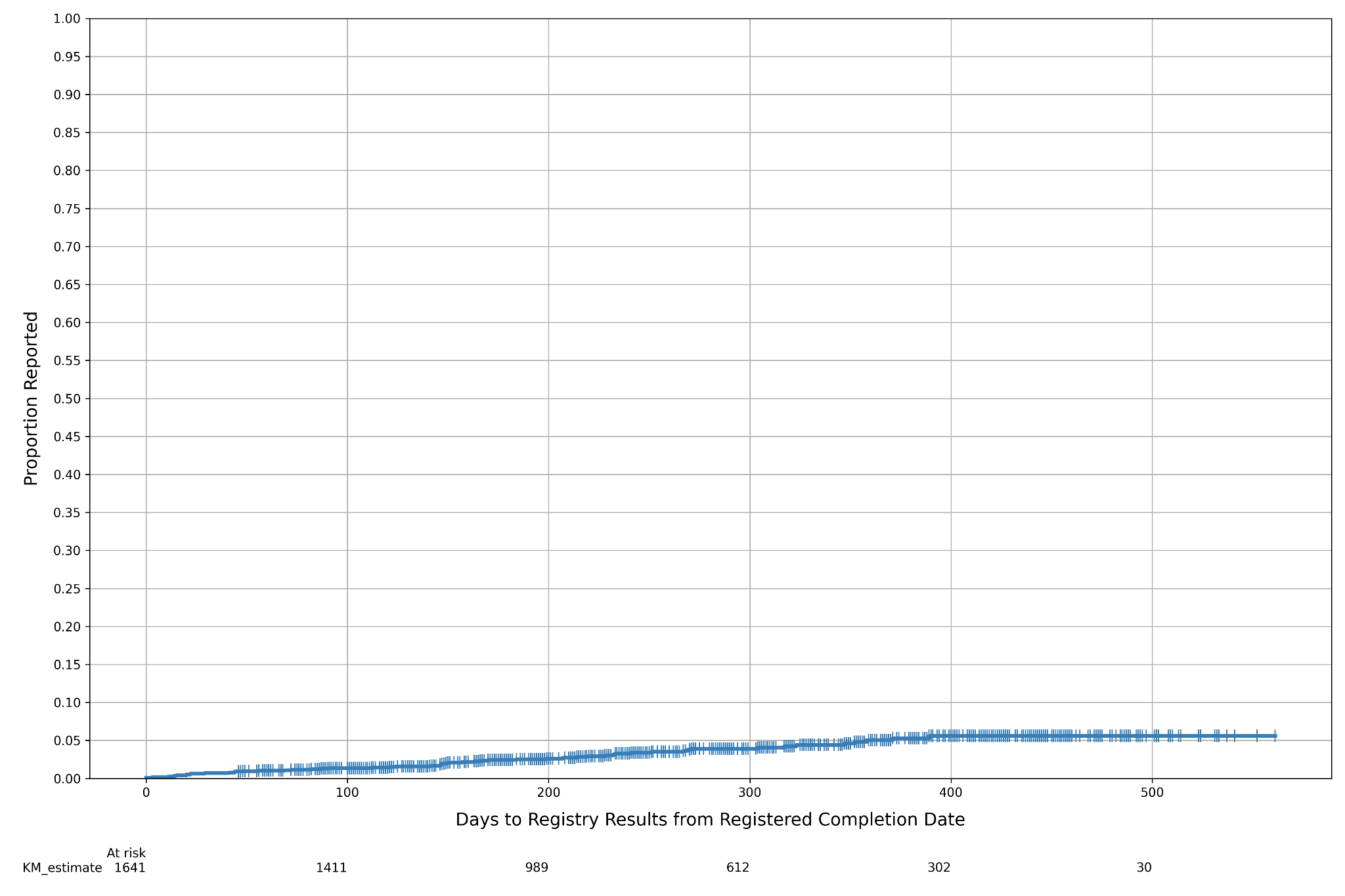
**

###### Supplemental Figure 2. Time to report for trials across all registries. The main analysis reported in the manuscript reports only those registries with mature results reporting infrastructure.

######

###### **Supplemental Table S2: Interventions Examined by COVID-19 Clinical Trials**

| **Intervention** | **Number of Trials** |
| --- | --- |
| Traditional Medicine | 205 |
| Hydroxychloroquine | 138 |
| Convalescent Plasma | 118 |
| Stem Cells (Mesenchymal) | 50 |
| Azithromycin | 46 |
| Ivermectin | 46 |
| Tocilizumab | 41 |
| Ritonavir | 40 |
| Favipiravir | 38 |
| Lopinavir | 33 |
| Chloroquine | 31 |
| Heparin (LMWH or Unfractionated) | 26 |
| Povidone-iodine | 22 |
| Methylprednisolone | 21 |
| Vitamin D (Any) | 21 |
| Nitazoxanide | 20 |
| Colchicine | 19 |
| Vitamin C | 19 |
| Immunoglobulin | 18 |
| Dexamethasone | 17 |
| Remdesivir | 17 |
| Prone Positioning | 14 |
| Anakinra | 13 |
| BCG Vaccine | 13 |
| Ruxolitinib | 13 |
| Zinc (Any) | 13 |
| Interferon Alpha (Any) | 11 |
| Hydrogen peroxide | 10 |
| Interferon Beta (Any) | 10 |
| Sofosbuvir | 10 |

###### Supplemental Table 1. Number of trials investigating top 30 most common interventions. Traditional medicine trials were not disaggregated by intervention and were therefore not included in our analysis.

######

######

###### **Supplemental Figures S3A-D: Cumulative Incidence Curves for Sensitivity Analyses**

**A)**

*
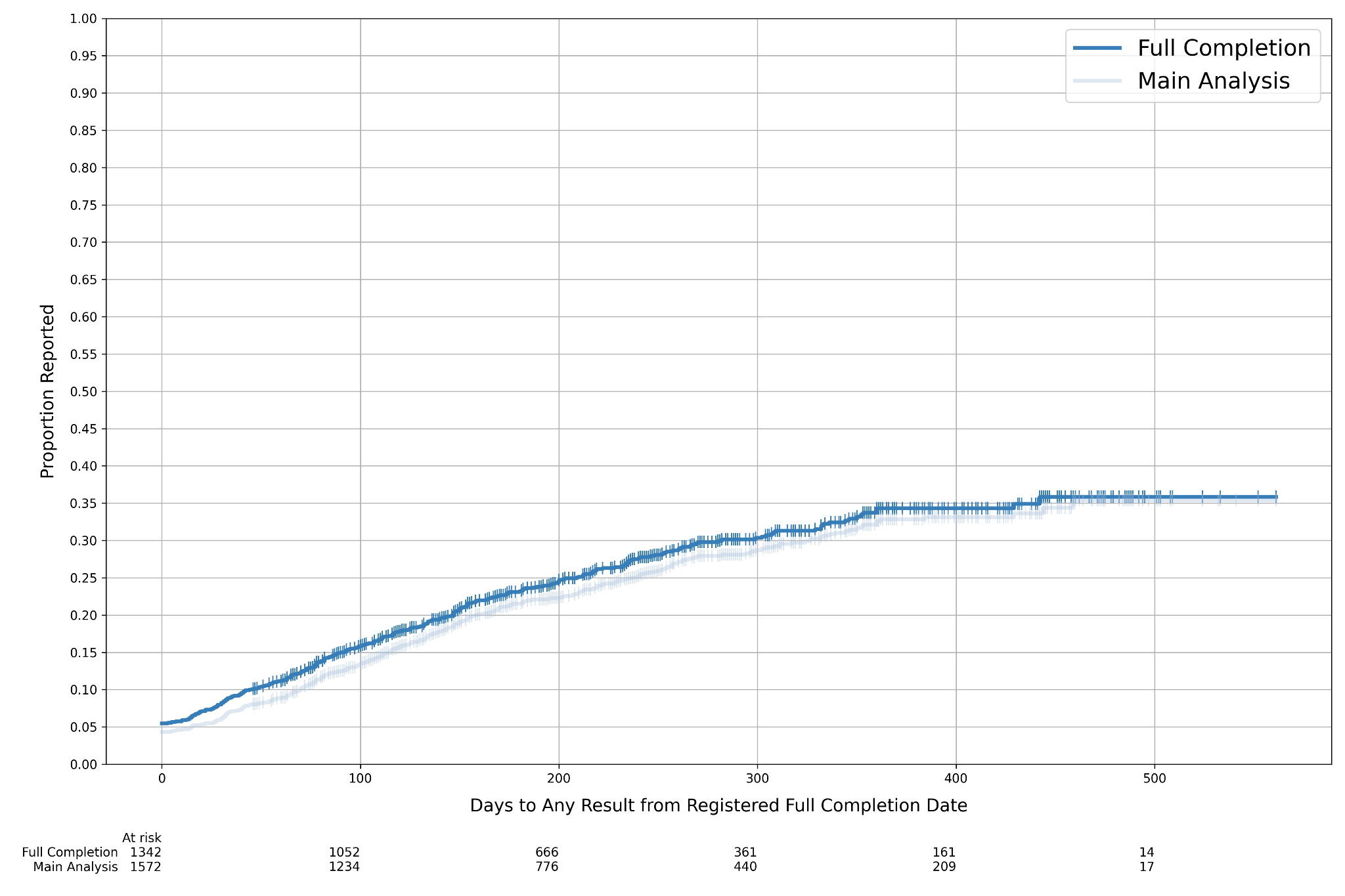
*

**B)**

*
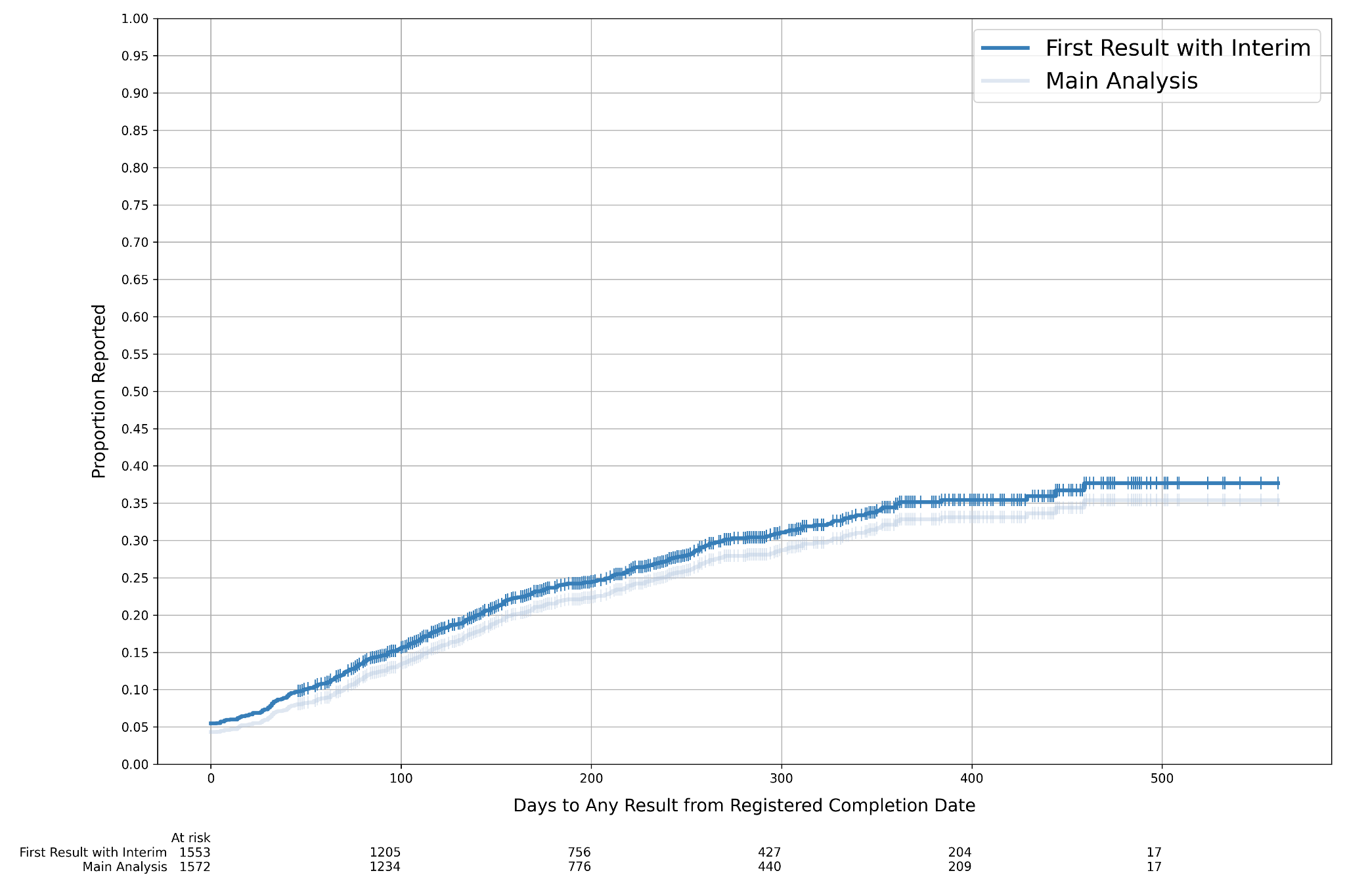
*

**C)**

######
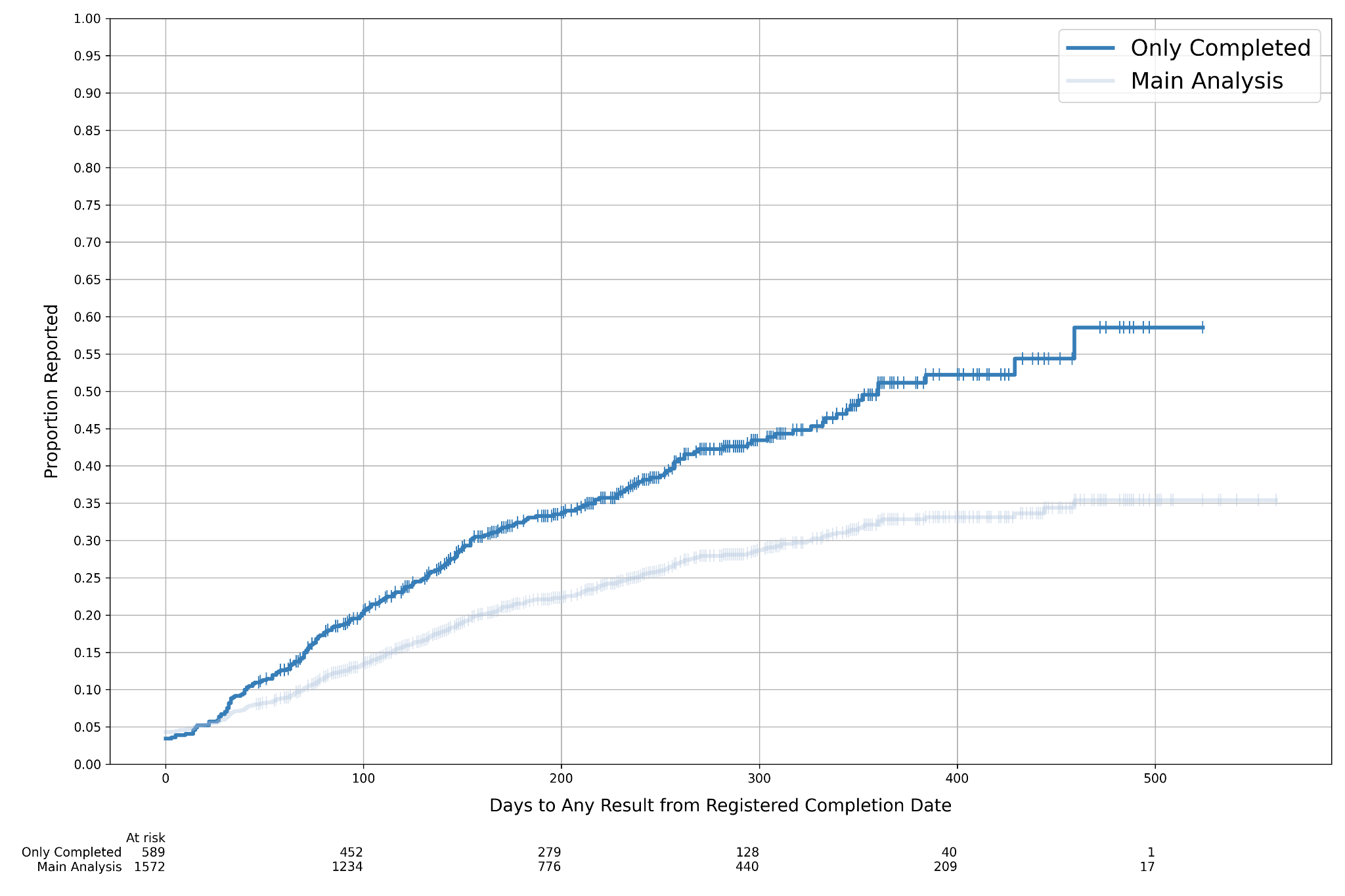


**D)**
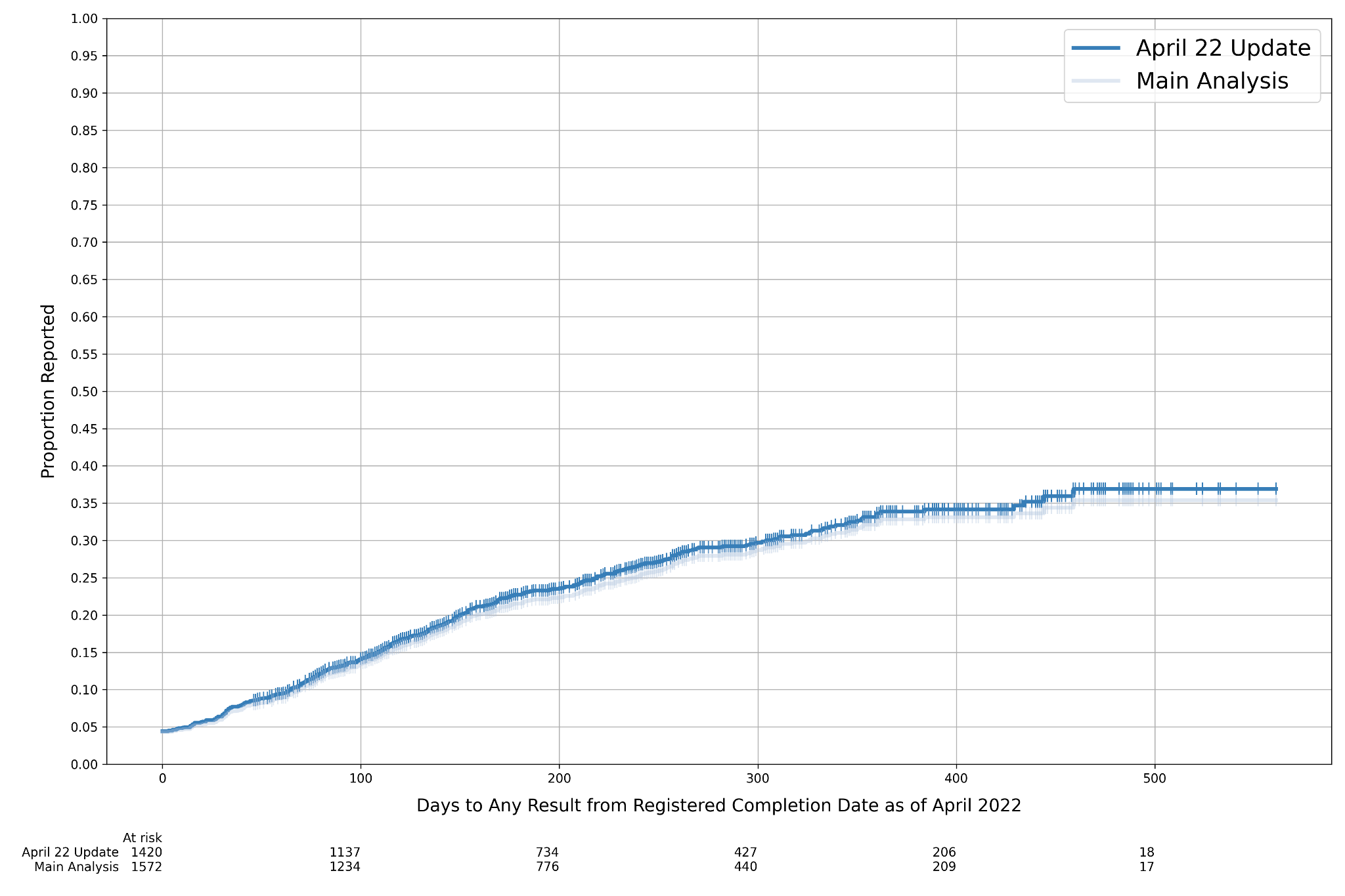


###### Supplemental Figure 3. (A-D) Time to dissemination for sensitivity analyses. (a) Trials that reached full study completion date. (b) Trials with a “completed” status. (c) Including interim results. (d) Trials using registry data in April 2022.
